# Determinants of Modern Contraceptive Methods Discontinuation among Women within Reproductive Age in Dire Dawa City, Eastern Ethiopia

**DOI:** 10.1155/2020/3059435

**Published:** 2020-07-30

**Authors:** Gizaw T. Yifru, Mesfin T. Haileyesus, Belay Tafa Regassa

**Affiliations:** ^1^Department of Statistics, College of Natural and Computational Sciences, Dire Dawa University, P.O. Box 1362, Dire Dawa City Administration, Ethiopia; ^2^Department of Micro Biology, College of Mediceneand Health Sciences, Ambo University, P.O. Box 19, Ethiopia

## Abstract

**Background:**

Modern family planning methods are widely believed to influence fertility reduction worldwide. Family planning had a clear effect on the health of women, children, and families worldwide especially those in developing countries. It has been shown that there are many instances in which women might discontinue contraception methods that put women's health at risk.

**Objectives:**

To assess and identify Determinants of Modern Contraceptive Methods Discontinuation among Women in Reproductive age interval in Dire Dawa City.

**Method:**

A cross-sectional study design was employed. A total of 811 respondent women with one-year history of modern contraceptive method usage were considered in the study. A stratified random sampling method was used to select the study participants. Data was collected using a structured questionnaire and analyzed by descriptive statistics and binary logistic regression.

**Result:**

The study indicated that 634 (78.20%) of respondent mothers continued using the method that they have used before a year. Whereas 177 (21.80%) of women discontinued using the method within a year. The factors age, number of children, who made the decision on the choice of the method used, the type of contraceptive method used, and taking counseling before using the method were found significant at 5% level of significance.

**Conclusion:**

Young women, respondents who have no or a small number of children, and not the decision maker on the choice of the method were more likely to discontinue. Whereas women who did not take counseling are less likely to discontinue. When compared to women who used implant those women who used pills and injectables are more likely to discontinue. Thus, the study identified factors that contribute to the discontinuation of modern contraception methods.

## 1. Introduction

The World Health Organization (WHO) estimated that 210 million women get pregnant each year and that about two-thirds deliver live infants globally. The remaining one-third of pregnancies ends in still births, miscarriages, and induced abortions [1].

According to the 2016 Ethiopia Demographic and Health Survey (EDHS), fertility in Ethiopia has declined from an average of 5.4 children per woman in 2005 to 4.8 children per woman in 2011 and 4.6 children per woman in 2016. The survey also revealed that in 2016, the total fertility rate (TFR) is 4.6 children per woman. However, the total wanted fertility rate is 3.6 children per woman.

Modern family planning methods are widely believed to influence fertility reduction worldwide. Family planning had a clear effect on the health of women, children, and families worldwide especially those in developing countries [2, 3].

Contraceptive prevalence at the global level will need to be at least 66%–75% in the more developed regions and 67% in the less developed regions to attain the projected decline in fertility by the year 2025 [4]. Regarding modern contraceptive method, the 2016 Ethiopia Demographic and Health Survey (EDHS) revealed that Modern contraceptive use by currently married Ethiopian women has steadily increased over the last 15 years, 6% of women using modern contraceptive method in 2000, 14% in 2005, 27% in 2011, and 35% in 2016. The survey also showed that in Dire Dawa among married women, the contraceptive prevalence rate is 29%. This figure seems fair compared to the lowest 1% in the Somali region and the highest 50% in Addis Ababa. However, all of the regions did not meet the expected 66% prevalence rate set by the Ethiopian Ministry of Health in 2011 [5].

More than one-third of all contraceptive users (35%) discontinued use within 12 months. The most common reason for stopping a method was the desire to become pregnant (42%), followed by method-related health concerns or side effects (18%). Discontinuation rates are highest for the pill (70%) followed by injectables (38%), IUD (13%), and lowest for implant (11%) [6].

In countries with moderate to high contraceptive prevalence, the majority of unintended pregnancies are the result of contraceptive discontinuation or failure [7]. Contraceptive discontinuation is a public health concern because of its association with negative reproductive health outcomes. Discontinuation rate is increasing with a remarkable figure in Ethiopia. But reasons for discontinuing the method were not well addressed in different studies within the country. Dynamics of contraceptive use, continuation, switching, and failure are important markers of how well programs are meeting the FP needs of women and couples. Studying the dynamics of contraceptive use can reveal problems in the use of contraceptive technologies and the gaps in the provision of services and, therefore, provide guidance essential for improving services is very important [8].

There are several studies conducted on the socioeconomic, cultural, and physical barriers women have to overcome in order to adopt a modern contraceptive method. However, somewhat less attention is given to what happens after a woman has overcome these barriers and adopts a method [9]. In addition, it has been shown that there are many instances in which women might discontinue contraception or switch methods that put women at risk of unwanted pregnancies [10]. Therefore, to decrease the prevalence of unmet need of contraception, one must uncover the extent to which and the reasons why contraceptive users become nonusers. Therefore, the purpose of this study was to assess and identify determinants of reversible modern contraceptive methods discontinuation among women of reproductive age group in Dire Dawa city, Eastern Ethiopia.

## 2. Material and Methods

### 2.1. Study Area

The study was conducted in Dire Dawa city. Dire Dawa city is located in the eastern part of Ethiopia, around 517 km to the east of the capital Addis Ababa. The city is one of the oldest cities that established as a result of the start of the former Ethio-France rail way transportation company in the year 1903.

### 2.2. Study Dataset

The study used part of the original dataset collected by the researchers with a fund obtained from Dire Dawa University. The data was collected between January-March 2017. Since the study is about discontinuation experiences of MCM user women, we sampled women with one year experience of using MCM at the time of survey. An eligible sample was selected by asking screening questions.

### 2.3. About the Original Dataset

The original dataset was a cross-sectional data that contain five years (2012-2017) histories of women who use MCM. A stratified random sampling technique was used to select the study participants. There are 9 kebeles in Dire Dawa city. Thus, the sample was taken from each kebeles, and proportional allocation was used to set the required total number of respondents from each kebeles. The sample size was determined in such a way that first, the approximate total population size (modern contraceptive methods user women) was taken from the Dire Dawa regional health bureau that was 21500 user women in 2016. The approximate total modern contraceptive methods (MCM) user women were the sum of users in each one of the 9 kebeles in Dire Dawa city. Then, the sample size formula stipulated in [11] was used to get the desired sample size. Since the researchers had no preliminary information on the values of the population proportion of women who discontinue MCM (P) and its complement *Q* = 1 − *P*, a pilot survey on 40 MCM user women was conducted and the researchers used the results to approximate the values as *P* = 0.32 and *Q* = 0.68. The level of significance *α* = 0.05 and allowable margin of error (*d* = 0.03). Then, the sample size became 929.

The sample unit was obtained by using a systematic sampling technique. That is the study participants' household was selected through systematic sampling technique at every “𝑘^th^” interval, whereas the first household was selected by lottery method, then continuing to every 𝑘^th^ household; if there was no respondent in the household, the collection continued to the next house until the desired sample size was attained.

An interviewer-administered questionnaire that addresses several issues related to factors of discontinue using MCM was prepared from related studies and administered to the sampled respondents. A pilot test was carried out to test the validity and reliability of the questionnaire and some correction was taken. Eighteen well-trained female health workers with three supervisors including the researchers were assigned to collect the required data through face to face interviews. Before data collection, data collectors explained the purpose of conducting this research, and consent was granted from the participants.

Approval and letter of permission were obtained from Research and Technology Interchange Affairs Directorate of Dire Dawa University and Dire Dawa Administration Health Bureau before the commencement of the study. Informed consent was sought and obtained from each participant before the commencement of this study. In order to ensure the confidentiality of the information, all data were kept in secret and coded in anonymity.

### 2.4. Variables of the Study

The dependent variable of the study was modern contraceptive methods utilization status (*y* = 0, if a woman has been using the method she using before 12 months and *y* = 1, if a woman discontinued the method she used a year ago due to experienced side effects).

The independent variables were women's age, marital status, educational level, average monthly income in Ethiopian Birr, number of children, and time taken to travel from home to the nearest health center to get family planning services. Types of modern contraceptive method used, ultimate method choice decision made (not by the user woman, by the user woman), and taking training/counseling before using the method (no, yes).

### 2.5. Data Analysis

Descriptive statistics tools such as frequency and percentage were used to present the collected data and to point out special features. Chi-square test was employed to test whether there are statistically significant association between MCM utilization status and independent factors. The classical Binary Logistic Regression model was fitted to identify significant predictor variables. Statistical package for Social Sciences (SPSS) version 22 was used.

## 3. Results

As mentioned earlier, this study used the information on a MCM used by women at a year before the time of the survey. At a year before the time of the survey, out of the total 929 women found in the original dataset, 14 of the women were found to be pregnant due to method failure, 18 discontinued due to infecundity, and 86 of the women discontinued due to a desire to get pregnant. Thus, the analysis was carried out using the remaining 811 respondents.

Out of the total study women, 634 (78.20%) of them continued using the method that they used before one year without changing. However, 177 (21.82%) of women discontinued using the method that they used before a year ago.

The main reason for contraceptive discontinuation were fear of experienced side effects of the method used (27.12%), want more effective method (18.64%), fear of side effect that may cause(15.25%), fear of infertility (12.99%), health worker recommendation(10.70%), and husband influence (7.90%) of the reasons (see Table 1).

Regarding age, 101 (12.5%) were aged less than 25 years; in this age group, out of the 101 women, 58 (57.4%) of them discontinue using the method within a year. Whereas 219 (27%) of the respondents were at least 35 years old, and out of the 219 women, 36 (16.4%) of them discontinue using the method within one year (see Table 2).

Regarding educational level, 54 (6.7%) respondents have no formal education, 51 (6.3%) respondents were able to read and writing, 219 (27%) respondents achieved primary level education, 248 (30.6%) respondents achieved secondary level education, and the remaining 239 (29.5%) respondents have a college diploma or above. The majority of women, 725 (89.4%) of them were married (see Table 2).

Out of the total study subjects, 61 (7.5%) of women have no child, 641 (79.0%) of women have 1-3 children, and the remaining 109 (13.4%) of women have more than 3 children. Out of the 61 women who have no child, 39 (63.9%) of them discontinued using the method within a year. Whereas out of the 109 women who have more than 3 children, only 13 (11.9%) of them discontinued using the method within a year (see Table 2).

Out of the total study women, 752 (92.7%) of them made the ultimate decision on method choice. Whereas 59 (7.3%) of them were not the ones who decide the method choice. Among the 59 women who were not the decision maker, 34 (57.6%) of them discontinue using the method within a year (see Table 2).

Out of the total study subjects, 169 (20.8%) of women used pills; out of the 169 pill user women, 73 (43.2%) of them discontinued using the pills. Out of the total study participants, 367 (45.3%) of women used injectables, out of these 116 (31.6%) of women discontinued using the injectables. Whereas out of the 229 (28.2%) women who used implant, only 35 (15.3%) of them discontinued using the method within a year (see Table 2).

Out of the total study participants, 579 (71.4%) of women took training/counseling before they used the method. Out of these 579 women who took training/counseling before they used the method, 189 (32.6%) of them discontinued using the method within a year. Regarding time taken to the nearest family planning center, slight differences on utilization status were observed (see Table 2).

Before proceeding to model fitting that contains several (multiple) independent variables, one should first check the significance of incorporating each one of the independent variables. If a given independent variable is insignificant in univariate analysis, then it is unnecessary to incorporate the variable in model fitting that contains multiple independent variables. Since the dependent variable of this study modern contraceptive method utilization status was a dichotomous variable, here in addition to univariate analysis, to incorporate more variables less conservative chi-square test with a 10% level of significance was employed. Chi-square test of association was employed to test whether there are statistically significant association between MCM utilization status and each of the independent variables. Independent variables that are found to be significant in the chi-square test were included in the Binary Logistic Regression model.

The results of the chi-square test showed that variables such as age, number of children, who made the decision on the choice of the used method, the type of contraceptive method used, and taking training/counseling before using the method were found to be significantly associated with modern contraceptive utilization status. However, no statistically significant association was found between modern contraceptive utilization status and variables such as educational level, marital status, time taken from home to nearest health center, and average monthly income (see Table 3).

Binary Logistic Regression model was fitted to identify significant predictor variables. All independent variables that were found to be significantly associated with contraceptive utilization status in the chi-square test were included in the model.

The results showed that variables such as age, number of children, who made the decision on the choice of the used method, the type of contraceptive method used and taking training/counseling before using the method were found to be significantly associated with modern contraceptive utilization status at 5% level of significance (see Table 4).

The estimated odd ratio of woman who is less than 25 years old is 4.664, implying that the risk of discontinuation of modern hormonal contraceptive method for a woman who is less than 25 years old is 66.4% higher than a woman who is at least 35 years old controlling for the other covariates in the model. The estimated odd ratio of a woman whose age is between 25-34 years old is 1.676, implying that the risk of discontinuation of a modern contraceptive method for a woman whose age is between 25-34 years old is 67.6% higher than a woman who is at least 35 years old controlling for the other covariates in the model (see Table 4).

The estimated odd ratio of a woman who has no child is 4.953. This shows that a woman who has no child has a 95.3% higher risk of discontinuation of modern contraceptive methods than women who has more than three children (reference group) controlling for other covariates in the model (see Table 4).

The estimated odd ratio of a woman who did not make a decision on the choice of the method is 3.47. This shows that the risk of MCM discontinuation for a woman who did not make a decision on the choice of the method is 3.47 times more likely than a woman who made a decision method choice controlling for other covariates in the model (see Table 4).

The estimated odd ratio of a woman who used pills is 4.106, implying that the risk of contraceptive discontinuation for a woman who used pills is 10.6% higher than a woman who used implant (reference group) controlling for other covariates in the model. The estimated odd ratio of a woman who used injectables is 2.179, implying that the risk of contraceptive discontinuation for a woman who used injectables is 17.9% higher than a woman who used implant controlling for other covariates in the model (see Table 4).

The estimated odd ratio of a woman who did not take training/counseling before taking the method is 0.547, implying that the risk of discontinuation of modern contraceptive method for a woman who did not take training/counseling before taking the method is 45.7% less likely than a woman who took training/counseling before taking the method controlling for the other covariates in the model.

## 4. Discussions

The study found that variables age, number of children, decision maker on the choice of the used method, the type of contraceptive method used, and taking counseling before using the method were significantly associated with the discontinuation of modern contraceptive method uses. Whereas explanatory variables: educational level, marital status, time taken from home to the nearest health center, and average monthly income were found to be statistically insignificant. The mothers with counseling were likely to discontinue MCM. Regarding the variables that are found to be insignificant, the result of this study contradicts with the finding of the study conducted in Jimma, Southwest Ethiopia, that found marital status and educational status are significant predictors of contraceptive discontinuation [12].

The result also disagrees with a finding of a study conducted in Northern India that found time taken from home to the nearest family planning center is significantly associated with contraceptive discontinuation [14].

Our study signaled that the risk of discontinuation of a modern contraceptive method for a younger woman is higher than the old woman who is at least 35 years old. This result contradicts with studies that showed women who discontinued are largely concentrated between the ages of 25-34 years [12, 13].

The study also showed that the risk of discontinuation for a woman who used pills or injectables is higher than a woman who used implant. Similar results also found in a study conducted in Ghana that showed that Pill users were more likely to discontinued [9].

The study found that the risk of discontinuation of modern contraceptive method for woman who have no child or who have 1-3 children are higher than a woman who has more than 3 children. This result agrees with a study found that women with many children would be less likely to stop using than women with few children [9].

Our study revealed that the risk of MCM discontinuation for a woman who did not make a decision on the choice of the contraceptive method higher. This finding is in agreement with [8].

The study also found that the risk of discontinuation who did not take counseling before taking the method is less likely than a woman who took counseling before taking the method. This could be due to women who did not take training/counseling before taking the method are less likely to look for alternatives. On an others side, as reviewed studies from Niger and Gambia, those women who felt that they had not properly counseled were more likely to discontinue using [15]. There was a significant association between going for counseling and education. Thus, they may oblige to switch if they experience side effects after counseling.

## 5. Conclusions and Recommendations

The findings of the study implied that explanatory variables age, number of children, who made the decision on the choice of the MC method used, the type of contraceptive method used, and taking counseling before using the method were significantly associated with the discontinuation of modern contraceptive methods. Women who are young were more likely to discontinue; women who have no child or less number of children were more likely to discontinue compared to women who have more than three children. Women who did not make a decision on the choice of the contraceptive method are more likely to discontinue. Surprisingly, women who did not take counseling were less likely to discontinue compared to women who have taken training and counseling. In comparison to women who responded as they used implants those women who responded they used pills and injectables were more likely to discontinue.

Therefore, based on the study findings and in order to reduce the discontinuation of MCM use, we forward the following action plans to be taken by the respective concerned body in the city. The significance of the type of method used may indicate that a woman did not get her choice due to healthcare worker influence, and lack of access to the preferred method. Health care worker bias occurs when they think that they are better aware of the method choose and the most appropriate method for their client, or if a woman shared provider decision and she does not understand why she is using a particular method. Thus, in family planning intervention effort needs to be exerted to reduce provider bias via delivering an appropriate training and counseling to health workers. High discontinuation in pills and injectable users may show that women seek a long term contraceptive methods which is the goal of the family planning program. Thus, as a remedial action, its better if family planning providers make sure that all methods are easily available. Training and counseling was a negatively associated with MCM uses continuation. Thus, the working training and counseling service must be improved for the better effectiveness of the program in Dire Dawa City.

## Figures and Tables

**Table 1:** Reason for discontinuation of MCM.

Reasons for discontinuation of MCM	Frequency	Percentage
Fear of infertility	23	12.99
Fear of method failure	4	2.26
Husband influence	14	7.90
Health worker recommendation	19	10.70
Fear of experienced side effects	48	27.12
Fear of side effect may cause	27	15.25
Want more effective method	33	18.64
Health-related problem	6	3.39
Religion influence	3	1.69
Total	177	100

**Table 2:** Descriptive summary of variables of the study.

Independent variables	Frequency (%)	MCM utilization status	
		Continued	Discontinued
		Frequency (%)	Frequency (%)
Age in years			
<25	101 (12.5)	43 (42.6)	58 (57.4)
25-34	491 (60.5)	348 (70.9)	143 (29.1)
≥35	219 (27.0)	183 (83.6)	36 (16.4)
Educational level			
No education	54 (6.7)	43 (79.6)	11 (20.4)
Read and write	51 (6.3)	41 (80.4)	10 (19.6)
Primary	219 (27.0)	149 (68.0)	70 (32.0)
Secondary	248 (30.6)	163 (67.7)	80 (32.3)
Diploma/above	239 (29.5)	173 (72.4)	66 (27.6)
Marital status			
Not married	86 (10.6)	55 (64.0)	31 (36.0)
Married	725 (89.4)	519 (71.6)	206 (28.4)
Monthly income			
<2000 birr	659 (81.3)	465 (70.6)	194 (29.4)
2000-4000	96 (11.8)	71 (74.0)	25(26.0)
>4000 birr	56 (6.9)	38 (67.9)	18(32.1)
No. of children			
0	61 (7.5)	22 (36.1)	39 (63.9)
1-3	641 (79.0)	456 (71.1)	185 (28.9)
>3	109 (13.4)	96 (88.1)	13 (11.9)
Method chose			
Not by the user	59 (7.3)	25 (42.4)	34 (57.6)
By the user	752 (92.7)	549 (73.0)	203 (27.0)
Type of method			
Pills	169 (20.8)	96 (56.8)	73 (43.2)
Injectables	367(45.3)	251(68.4)	116 (31.6)
IUCD	46 (5.7)	33 (71.7)	13 (28.3)
Implant	229 (28.2)	194 (84.7)	35 (15.3)
Training/counseling			
No	232 (28.6)	184 (79.3)	48 (20.7)
Yes	579 (71.4)	390 (67.4)	189 (32.6)
Time taken			
<10minute	88 (10.9)	62 (70.5)	26 (29.5)
10-20	586 (72.3)	420 (71.7)	166 (28.3)
>20minutes	137 (16.9)	92 (67.2)	45 (32.8)

**Table 3:** Summary result of the chi-square test.

Independent variables	Chi-square calculate	D.F.	*P* value
Age	56.149	2	<0.001^∗^
Educational level	6.524	4	0.163
Marital status	2.165	1	0.141
Monthly income	0.715	2	0.699
No. of children	51.341	2	<0.001^∗^
Method chose	24.820	1	<0.001^∗^
Type of method	38.494	3	<0.001^∗^
Time taken	1.101	2	0.577
Training/counseling	11.441	1	0.001^∗^

^∗^Significant at 0.05 level of significance.

**Table 4:** Results of the Binary Logistic Regression model.

Explanatory variables	*β*	SE	Exp(*β*)	*P* value
Age				
<25	1.540	0.306	4.664	<0.001^∗^
25-34	0.517	0.233	1.676	0.027^∗^
≥35			1	
No. of child				
0	1.600	0.441	4.953	<0.001^∗^
1-3	0.616	0.338	1.852	0.068
>3			1	
Method chose				
Not by the user	1.244	0.301	3.470	<0.001^∗^
By the user			1	
Type of method				
Pills	1.413	0.255	4.106	<0.001^∗^
Injectables	0.779	0.228	2.179	0.001^∗^
IUCD	0.650	0.410	1.915	0.113
Implant			1	
Get training				
No	-0.603	0.202	0.547	0.003^∗^
Yes			1	
Constant	-2.761	0.365		<0.001^∗^

^∗^Significant at 0.05 level of significance.

## Data Availability

The data to support the findings can be obtained from the corresponding author upon request.
